# Ideal Combinations of Acceleration-Based Intensity Metrics and Sensor Positions to Monitor Exercise Intensity under Different Types of Sports

**DOI:** 10.3390/s22072583

**Published:** 2022-03-28

**Authors:** Wei-Han Chen, Chun-Wei Chiang, Nicholas J. Fiolo, Philip X. Fuchs, Tzyy-Yuang Shiang

**Affiliations:** 1 Department of Athletic Performance, National Taiwan Normal University, Taipei City 106, Taiwan; gn01800083@gmail.com (W.-H.C.); jack30815@gmail.com (C.-W.C.); nicholas.j.fiolo@ntnu.edu.tw (N.J.F.); philip.fuchs@ntnu.edu.tw (P.X.F.); 2 Graduate Institute of Sports Equipment Technology, University of Taipei, Taipei City 111036, Taiwan; 3 Department of Sport and Exercise Science, University of Salzburg, 5400 Salzburg, Austria

**Keywords:** wearable electronic devices, acceleration, running, team sports, racquet sports

## Abstract

This study quantified the strength of the relationship between the percentage of heart rate reserve (%HRR) and two acceleration-based intensity metrics (AIMs) at three sensor-positions during three sport types (running, basketball, and badminton) under three intensity conditions (locomotion speeds). Fourteen participants (age: 24.9 ± 2.4 years) wore a chest strap HR monitor and placed three accelerometers at the left wrist (non-dominant), trunk, and right shank, respectively. The %HRR and two different AIMs (Player Load per minute [PL/min] and mean amplitude deviation [MAD]) during exercise were calculated. During running, both AIMs at the shank and PL at the wrist had strong correlations (*r* = 0.777–0.778) with %HRR; while other combinations were negligible to moderate (*r* = 0.065–0.451). For basketball, both AIMs at the shank had stronger correlations (*r* = 0.604–0.628) with %HRR than at wrist (*r* = 0.536–0.603) and trunk (*r* = 0.403–0.463) with %HRR. During badminton exercise, both AIMs at shank had stronger correlations (*r* = 0.782–0.793) with %HRR than those at wrist (*r* = 0.587–0.621) and MAD at trunk (*r* = 0.608) and trunk (*r* = 0.314). Wearing the sensor on the shank is an ideal position for both AIMs to monitor external intensity in running, basketball, and badminton, while the wrist and using PL-derived AIM seems to be the second ideal combination.

## 1. Introduction

The use of wearable technology is becoming increasingly integrated into fitness and athletics [1,2,3]. Wearable devices are often user-friendly and affordable [4], allowing for wide spread adoption. In practical application, wearable devices can be employed to monitor exercise intensity [5] and quantify the quantity and characteristics of physical activity [6]. Exercise intensity measurements are classified as internal (i.e., linked to an individual’s physiological stress response) and external (i.e., independent of the characteristics of the individual) [5]. Heart rate (HR) -based metrics are a common method to quantify internal intensity, in which the percentage of heart rate reserve (%HRR) is currently the most recommended for the determination of the aerobic exercise intensity [7]. Acceleration-based intensity metrics (AIMs) are a common method to quantify external intensity [5], provide objective information for coaches and athletes to quantify exercise intensity and volume to enhance training efficiency [8]. Two different AIM commonly used to estimate external intensity are Player Load per minute (PL/min) and mean amplitude deviation (MAD).

Player Load (PL) is a commonly used metric in the field and court sports to quantify external training load [8,9,10,11,12,13] and to derive an average external intensity measurement (PL/min) [9]. PL is a function of the sum of changes in acceleration (ΣΔ acceleration), as measured by triaxial accelerometers [14]. Specifically, PL is calculated as the square root of the sum of the squared rates of change in acceleration between each moment of a training session in each movement axis (x, y, and z), and it is represented in arbitrary units [12,14,15]. Previous research has confirmed the validity of PL as a training load metric [8,16,17,18]. However, most of the above studies put an accelerometer on the upper back to calculate PL. Liu et al. [8] found that PLs calculated from accelerometers at different body locations varied substantially in the strength of correlation to HR-based training impulse (TRIMP) (racket and non-racket hands: *r* = 0.570, 0.796; lower back: *r* = 0.843; racket and non-racket legs: *r* = 0.728, 0.836). Therefore, it is necessary to clarify the combined impact on sensor-position and movement type on PL calculations and determine the ideal sensor-positions for evaluating the exercise intensity in different sports.

MAD describes the mean value of the dynamic acceleration component, expressed as the typical distance of data points about the mean [19]. It is calculated from the resultant value of the measured tri-axial acceleration, which comprises both tri-axial dynamic components due to velocity changes and static components due to gravity [20,21]. The static component is removed from the analyzed time period (epoch) and the remaining dynamic component is rectified. Thus, the MAD value can be regarded as the mean of the revised acceleration signal autonomous of the static element within the epoch [21]. Researchers generally set the time period (epoch) as 5 s because it is considered to be adequate for reporting different activities [19,22]. This means that the computation of the MAD metric provides a measure of the intensity for every 5 s of data. One of the advantages of MAD is that it is not affected by different brands of accelerometers [23] or different sensor-positions (middle of the back, both sides of the hip) [20]. Studies showed that there is a strong correlation between MAD values and HR [19,23] as well as MAD values and oxygen consumption (VO_2_, ml/kg/min) [20] during slow walking to running, the accelerometer was placed at wrist [19,21], side of the hip [20,23], or back [20]. Different from PL, MAD mainly focuses on the estimation of physical activity intensity (HR, VO_2_) and determent of the MAD cut-points (unit: mg) for classifying the activity patterns ranging from sedentary behavior to walking and running [19,20,21,23]. The unit of the MAD is generally expressed as milligravity (mg) to ensure that the developed cut-points would be comparable with the prior findings in the literature [19,20,21,23]. The majority of MAD application has focused on its ability to estimate and discriminate intensity during bipedal locomotion in physical activity research. However, the applicability of MAD to estimate the exercise intensity of highly varied movement types, such as basketball (team sports) and badminton (racket sports), has yet to be determined.

Previous research is limited to investigations of only one AIM calculation method and usually with an accelerometer on a single body placement site. It is unknown which AIM calculation methodology and sensor-position is optimal for different sports demands. The purpose of this study was to determine the strength of correlations between internal intensity metric (%HRR) and two AIMs (PL/min and MAD) under different accelerometer positions and under different sporting tasks (running, basketball, and badminton). The findings of this study contribute to clarifying the ideal combination of AIMs and sensor-positions for monitoring exercise intensity under different sports and provide evidence-based references for coaches and scientists.

## 2. Materials and Methods

### 2.1. Participants

Fourteen recreationally active male participants (mean ± SD age: 24.9 ± 2.4 years, height: 1.77 ± 0.04 m, mass: 74.6 ± 6.9 kg, resting heart rate: 72.1 ± 11.1 bpm) participated in this study. All participants reported no neuromuscular, musculoskeletal, or cardiovascular contraindications to exercise within six months prior to the experiment. All participants were right arm dominant and used the right hand to hold the racket in all badminton tasks. The study protocol was approved by the Institutional Review Board of National Taiwan Normal University (Approval Number: 201912HM103; date: 2020/02/17), following the Declaration of Helsinki. Participants were informed of the benefits and risks of the investigation prior to signing an institutionally approved informed consent document to participate in the study.

### 2.2. Measurement

**Figure 1:**
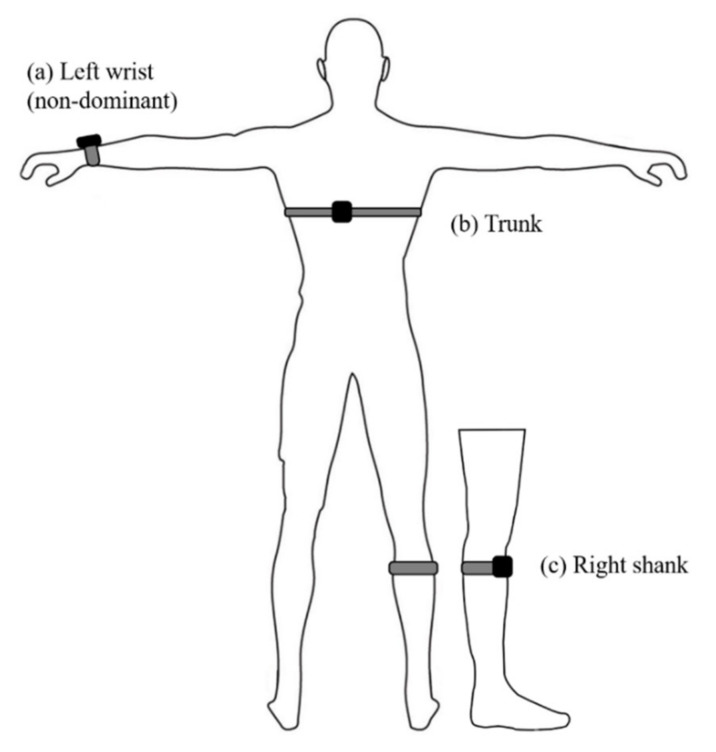
Accelerometers were placed on the left wrist (**a**), trunk (**b**) and shank (**c**).

A Polar H10 chest strap HR monitor (H10 Polar; Polar Electro. Oy, Kempele, Finland) was used in this study to record the HR at a sampling rate of 1 Hz. Three accelerometers (Naxsen 9, SIPPLink Co., Ltd., Hsinchu City, Taiwan) were used to record acceleration at a sampling rate of 200 Hz. The participant simultaneously wore three accelerometers at standardized body sites (non-dominant [left] wrist, trunk, and right shank), as shown in Figure 1. The trunk accelerometer was fixed to the heart rate belt and located on the left erector spinae muscle (T9~10) to avoid shaking. The shank accelerometer was fixed to the lateral of the tibial tuberosity. The shank accelerometer was placed on the right leg (racket-side) because the lunge with the racket-side leg is often performed in badminton [24]. This study did not place an accelerometer on the racket-wrist because the PL at this position was unable to estimate the internal load well alone due to the specificity of the racket motion to the racket hand [8].

### 2.3. Procedures

#### 2.3.1. Familiarization Trial

All participants completed a familiarization trial of all procedures prior to the data collection session.

#### 2.3.2. Experimental Procedure

Participants completed three data collection sessions on separate days. Data collection consisted of a running session, basketball session, and badminton session. Session order was randomly assigned. Each session consisted of a sport-specific task completed at 3 different intensities. Each task was performed for 3 min with a standardized recovery period between tasks. The subsequent task began once the participant’s HR decreased below 100 bpm. Task orders were randomized. Individual resting HR (HRrest) was assessed prior to testing on the first day of data collection. Participants sat on a chair for 3 min. The mean HR over the final minute of sitting rest was used to define individual HRrest.

#### 2.3.3. Running Session

In the running session, participants completed 3 min trials at 9, 12, 15 kph on a treadmill (Funa-7310, Tonic Fitness Technology Inc., Tainan Hsien, Taiwan). Treadmill trials were randomized.

#### 2.3.4. Basketball Session

**Figure 2:**
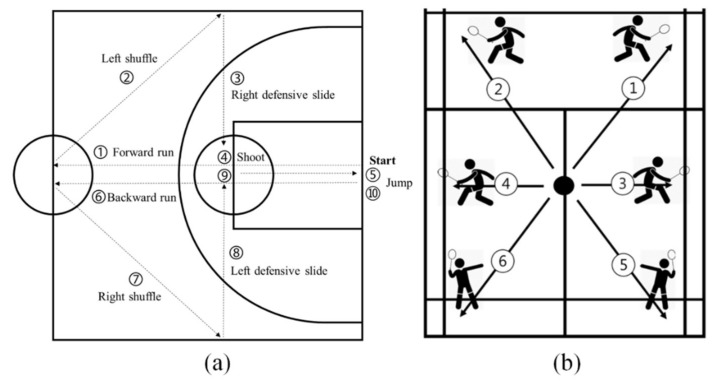
Custom basketball exercise routine (**a**) and badminton six-point footwork (**b**).

In the basketball session, a custom basketball exercise routine was completed to replicate the sport-specific demands of basketball. The routine consisted of 10 distinct tasks that involved a combination of running, shuffling, defensive slide, shooting, and jumping (Figure 2), those are common basketball movements [25]. The intensity was increased by decreasing the time allotted for completing each task (level 1: 7 s/task; level 2: 6 s/task; level 3: 5 s/task). The researcher read aloud the task’s time so that the participant can adjust the speed of the movement.

#### 2.3.5. Badminton Session

In the badminton session, participants performed six-point footwork (Figure 2) [8], which is a classic badminton training method. The routines were completed without shuttlecocks. Participants conducted sequentially the forehand and backhand net shot for task 1 and task 2, forehand and backhand swing for task 3 and task 4, and overhead swing for task 5 and task 6. Participants listened to the metronome beats to keep the movement tempo. Because the movement time required for task 3 and task 4 is short, both tasks were combined into one fluid transition task. As with the basketball session, the intensity was increased by decreasing the time allotted for each movement task (level 1: 5 s/task; level 2: 4.5 s/task; level 3: 4 s/task).

### 2.4. Data Analysis

This study analyzed the data of %HRR (Equation (1)), PL, and MAD during the third minute (121 to 180 s) [8] because the HR was relatively stable. A 10-Hz low-pass filter [26] was employed in acceleration data to eliminate high-frequency noises generated by vibration, and those data filtered were calculated to PL (Equation (2)) and MAD (Equation (3)). The calculated PL in this study is the accumulative PL for one minute, we named it PL/min to reflect the external intensity, as same as MAD. For the MAD analysis, the 5-s epoch duration was selected [19,27,28], and the mean of 12 successive 5-s epochs of MAD values was used for subsequent analysis.

(1) $$\%\mathrm{HRR}\;=\frac{\mathrm{HRex}-\mathrm{HRrest}}{\mathrm{HRmax}-\mathrm{HRrest}}\times 100$$

where HRex = average HR during exercise, HRrest = resting HR, and HRmax = maximum HR. The HRmax was predicted by using the equation of 208–0.7 × age, the equation has greater accuracy than the other equations studied for predicting observed values of HRmax in 18–25 years old adults [29].

(2) $$\mathrm{Player}\;\mathrm{Load}\;=\sum_{i}^{n}\sqrt{{\left(x_{i}-x_{i-1}\right)}^{2}+{\left(y_{i}-y_{i-1}\right)}^{2}+{\left(z_{i}-z_{i-1}\right)}^{2}}$$

$x_{i}$, $y_{i}$, and $z_{i}$ the *i*th acceleration for each axis (x, y, y) acceleration.

(3) $$\mathrm{MAD}\;=\frac{1}{n}\times \overset{n}{\underset{i=1}{\sum }}\left|\;r_{i\;}-\bar{r}\;\right|$$

MAD:$\;r_{i}$ the *i*th resultant acceleration within the epoch and $\bar{r}$ the mean resultant value of the epoch. epoch = 1000 samples (5 s). The unit of the MAD is milligravity (mg); ie, the Earth’s gravity of 1g is equal to 1000 mg.

### 2.5. Statistical Analysis

All statistical analyses were performed using IBM SPSS Statistics (version 23.0; IBM Corporation, Armonk, NY, USA). Normality of distribution was assessed via Shapiro-Wilk test, and selected accordingly statistical tests. Descriptive statistics were expressed in mean ± standard deviation. One-way repeated-measures ANOVA was employed to compare differences among three intensity levels at third minute data, reported as partial eta squared (η_p_^2^) as a measure of effect size and power (1−β). Mauchly’s test was employed to assess whether data were spherical, and applied the Greenhouse–Geisser correction when they were not. Paired t-tests with Bonferroni adjustment (multiply *p*-values by 3) were adopted for post hoc analysis. If the data was not normally distributed, the Friedman test was performed to compare differences among three intensity levels, reported as chi-square ($\chi_{\;}^{2}$) and degrees of freedom. Wilcoxon signed-rank test with Bonferroni adjustment (multiply *p*-values by 3) was used for post hoc analysis. The within-sport Pearson (*r*) and across-sports Spearman (*ρ*) correlation coefficient were employed to assess the relationship between PL/min and %HRR as well as MAD and %HRR. The correlation magnitude was evaluated on the basis of the following criteria: negligible: 0–0.09, weak: 0.10–0.39, moderate: 0.40–0.69, strong: 0.70–0.89, and very strong: 0.91–1.00 [30]. Statistical significance was set at *α* = 0.05.

## 3. Results

### 3.1. %HRR Intensity

**Figure 3:**
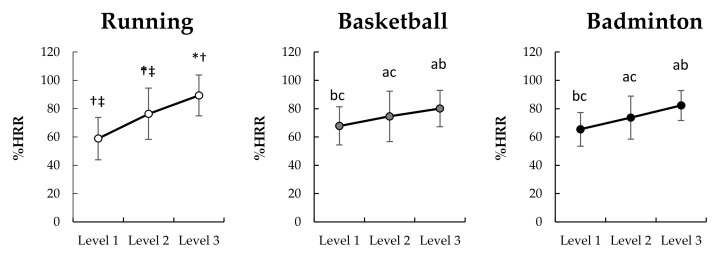
Change of %HRR among three exercise intensity levels under different sports (*n* = 14). Bonferroni post hoc test: significantly different than ^a^ Level 1, ^b^ Level 2, and ^c^ Level 3 (*p* < 0.05); Wilcoxon signed rank test: significantly different than * Level 1, ^†^ Level 2, and ^‡^ Level 3 (*p* < 0.05).

The results (Figure 3) showed that the %HRR under running ($\chi_{\left(2\right)}^{2}$ = 28, *p* < 0.001), basketball (F_(1.188,15.443)_ = 67.577, *p* < 0.001, η_p_^2^ = 0.839, 1−β = 1.000), and badminton (F_(2,26)_ = 12.409, *p* < 0.001, η_p_^2^ = 0.488, 1−β = 0.992) significantly increased with intensity levels.

### 3.2. Accelerometer Intensity

**Figure 4:**
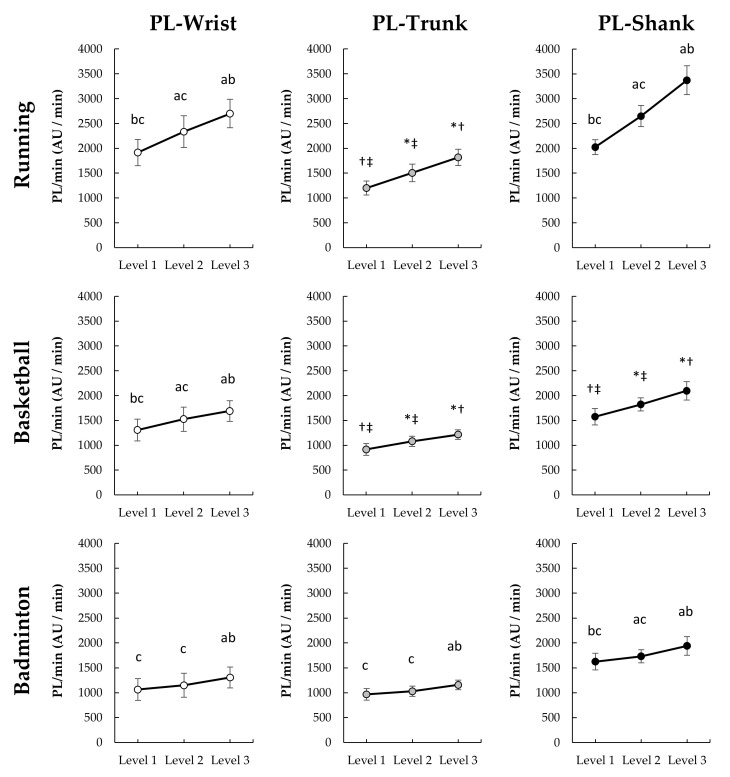
Change of PL/min among three exercise intensity levels under different sports (*n* = 14). Bonferroni post hoc test: significantly different than ^a^ Level 1, ^b^ Level 2, and ^c^ Level 3 (*p* < 0.05); Wilcoxon signed rank test: significantly different than * Level 1, ^†^ Level 2, and ^‡^ Level 3 (*p* < 0.05).

The PL/min at wrist (F_(2,26)_ = 121.809, *p* < 0.001, η_p_^2^ = 0.904, 1−β = 1.000), trunk ($\chi_{\left(2\right)}^{2}$ = 26.143, *p* < 0.001), and shank (F_(1.338,17.339)_ = 487.515, *p* < 0.001, η_p_^2^ = 0.974, 1−β = 1.000) under running significantly increased with intensity levels (*p* < 0.001, Figure 4). Under basketball, the PL/min at wrist (F_(2,26)_ = 71.733, *p* < 0.001, η_p_^2^ = 0.847, 1−β = 1.000), trunk ($\chi_{\left(2\right)}^{2}$ = 28.000, *p* < 0.001), and shank ($\chi_{\left(2\right)}^{2}$ = 28.000, *p* < 0.001) also significantly increased with intensity levels (*p* < 0.001). Under badminton, PL/min at the shank (F_(1.331,17.049)_ = 59.852, *p* < 0.01, η_p_^2^ = 0.822, 1−β = 1.000) also significantly increased with intensity levels (*p* < 0.01), but the PL/min at the wrist (F_(1.331,17.299)_ = 16.380, *p* < 0.001, η_p_^2^ = 0.558, 1−β = 0.988) and trunk (F_(1.249,16.241)_ = 18.345, *p* < 0.001, η_p_^2^ = 0.585, 1−β = 0.991) did not increased with intensity levels between level 1 and level 2 (wrist: *p* = 0.188, trunk: *p* = 0.390), the significant differences were found between level 1 and level 3 (wrist: *p* = 0.002, trunk: *p* < 0.001) and between level 2 and level 3 (wrist: *p* < 0.001., trunk: *p* = 0.000).

**Figure 5:**
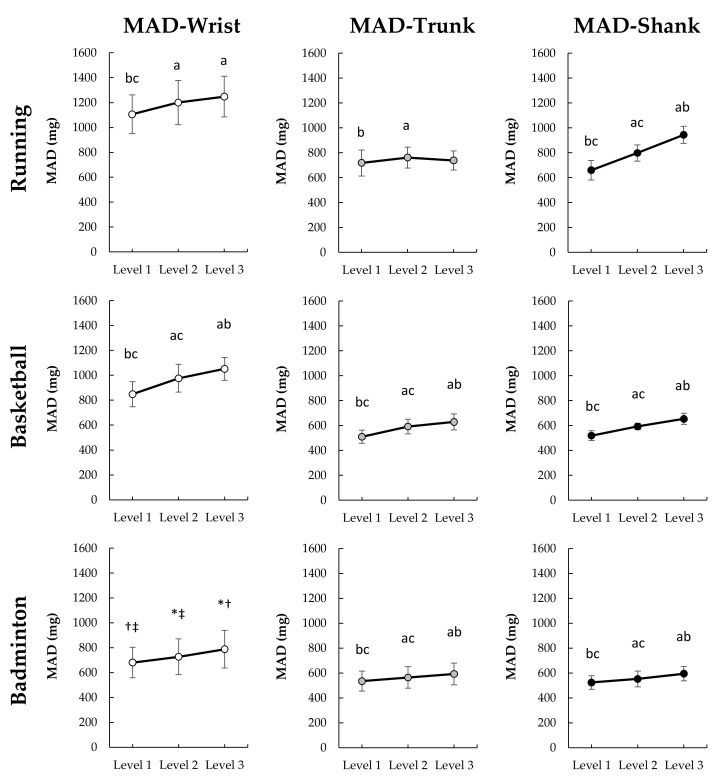
Change of MAD among three exercise intensity levels under different sports (*n* = 14). Bonferroni post hoc test: significantly different than ^a^ Level 1, ^b^ Level 2, and ^c^ Level 3 (*p* < 0.05); Wilcoxon signed rank test: significantly different than * Level 1, ^†^ Level 2, and ^‡^ Level 3 (*p* < 0.05).

The MAD at the wrist and shank under running (*p* < 0.001, η_p_^2^ = 0.657–914, 1−β = 1.000) significantly increased with intensity levels (Figure 5), but the MAD at wrist did not increased with intensity levels between level 3 and level 2 (*p* = 0.089), while the MAD at trunk (F_(2,26)_ = 4.868, *p* = 0.016, η_p_^2^ = 0.272, 1−β = 0.754) only significantly increased between intensity level 1 and level 2 (*p* = 0.008), no difference between level 1 and level 3 (*p* = 0.793) and between level 2 and level 3 (*p* = .202) were found. Under the basketball, the MAD at the wrist, trunk, and shank (F_(2,26)_ = 58.587–151.992, *p* < 0.001, η_p_^2^ = 0.818–921, 1−β = 1.000) significantly increased with intensity levels. The MAD on the wrist ($\chi_{\left(2\right)}^{2}$ = 19, *p* < 0.001), trunk (F_(2,26)_ = 30.178, *p* < 0.001, η_p_^2^ = 0.699, 1−β = 1.000) and shank (F_(2,26)_ = 41.169, *p* < 0.001, η_p_^2^ = 0.760, 1−β = 1.000) under badminton significantly increased with intensity levels.

### 3.3. Intensity Correlation by Sport and Sensor Location

**Figure 6:**
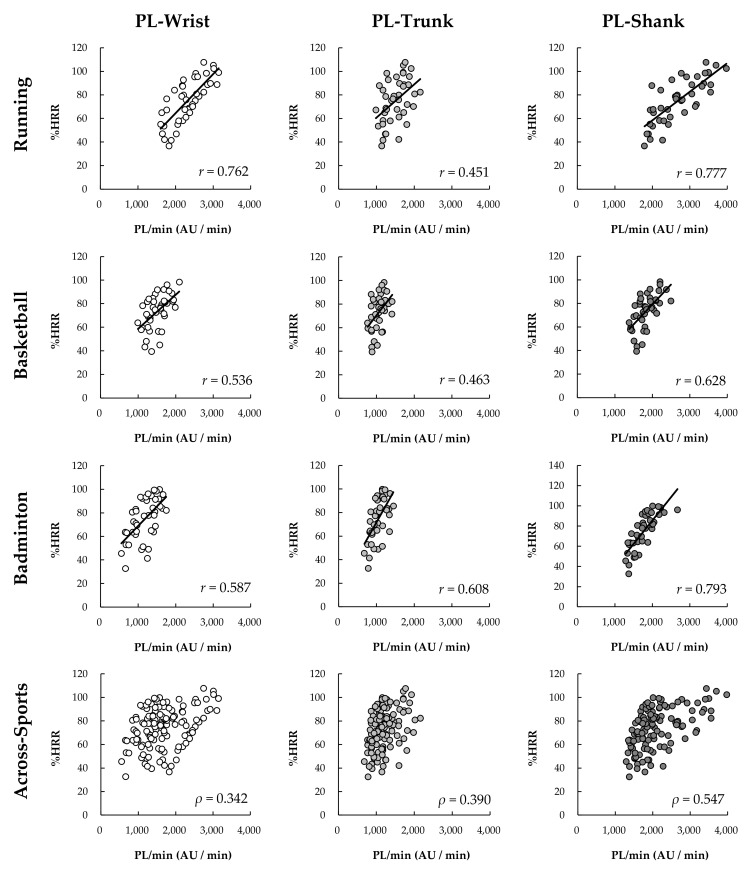
The within-sport Pearson (*r*) (*n* = 42) and across-sports Spearman (*ρ*) (*n* = 126) correlation coefficient for %HRR and PL.

**Figure 7:**
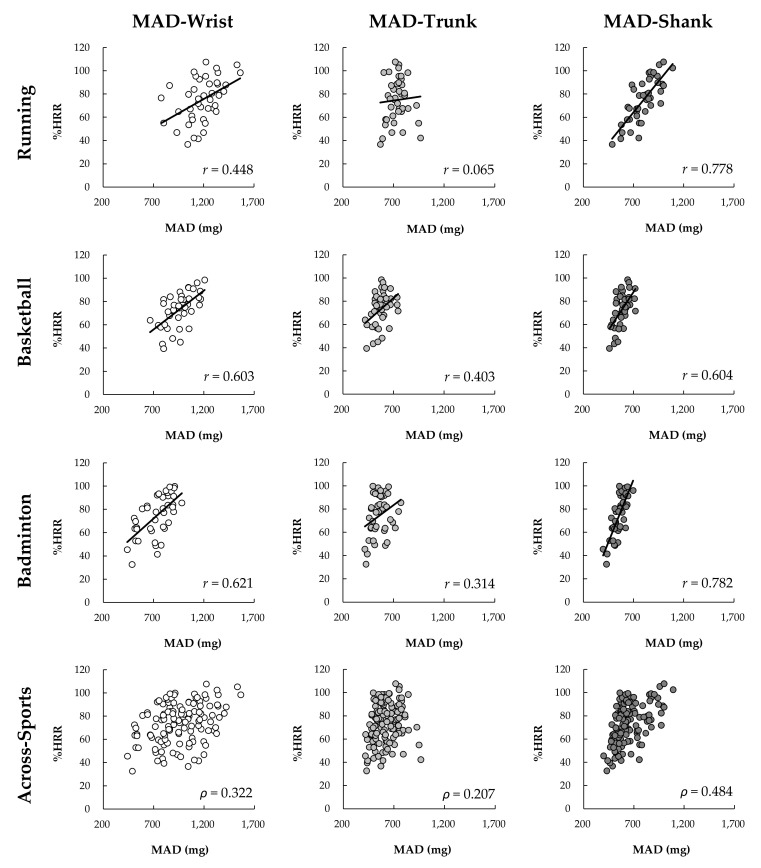
The within-sport Pearson (*r*) (*n* = 42) and across-sports Spearman (*ρ*) (*n* = 126) correlation coefficient for %HRR and MAD.

The results of within-sport correlation (Figure 6 and Figure 7) showed that PL and MAD at the wrist, trunk, and shank under three sports were significantly related to %HRR (*r* = 0.192–0.793, *p* < 0.05), except to the MAD at the trunk under running (*r* = 0.065, *p* = 0.684). The across-sports correlation results showed that PL and MAD at all sensor-placements were weak to moderate related to HR (*ρ* = 0.207–547, *p* < 0.05).

## 4. Discussion

The primary findings of this study were that both PL and MAD metrics are related to %HHR at a statistically significant level under different sports, regardless of sensor placement. The exception to this finding is MAD at the trunk for running. However, despite the statistical significance, the magnitude of the correlations varied substantially by the different AIM methods, sensor-placements, and sport types. For running, both AIMs at the shank (*r* = 0.777–0.778) and PL at the wrist (*r* = 0.762) had strong correlations with %HRR; however, moderate correlations were found in PL at the trunk and MAD at the wrist, while negligible correlations were found in MAD at the trunk (*r* = 0.065). In the basketball tasks, both AIMs at all placement sites produced moderate correlations with %HRR (*r* = 0.403–0.628). For badminton, both AIMs at shank placement produced the strongest correlations (*r* = 0.782–0.793), followed by the wrist and MAD at the trunk (*r* = 0.587–0.621), and finally the MAD at the trunk (*r* = 0.314). However, across-sports correlations between %HRR and both AIMs on all placements were weak to moderate. If considering the overall correlations with %HRR under different sports and across sports, wearing the sensor on the shank seems to be the most ideal placement regardless of AIMs, followed by the wrist and using PL-derived AIM seems to be the second ideal combination.

In the present study, PL and MAD at the wrist and shank had stronger correlations with %HRR than those correlations of both at the trunk (T9-10). This difference indicates that AIMs on the trunk may lack the sensitivity to capture the relationship between external intensity (acceleration) and internal intensity (HR). This inability could be because the cushioning of the lower limbs and waist reduce the variation in acceleration measured at the upper back. Additionally, the landing strategies and movement techniques employed may further reduce the variation in acceleration. We found that the MAD measurements at the trunk failed to increase with running speed from 12 to 15 kph (Figure 5), resulting in a negligible relationship between MAD and %HRR. This finding provides information gaps unknown in previous research. Vähä-Ypyä, Vasankari, Husu, Mänttäri, Vuorimaa, Suni and Sievänen [20] demonstrated the MAD on mid-trunk placements had very large within-subject correlations with intensity as measured by HR and VO_2_ during a walk to running at 2.2 to 10.8 kph. However, we found that MAD-based intensity measures on the trunk will produce biased and thus cannot be used to estimate intensity. Therefore, the combination of MAD methodology and trunk placement (upper-back) is unsuitable for monitoring intensity during fast running (>12 kph). Unlike MAD, PL-based intensity did systematically change with the increase of speed. However, the correlation with %HRR was moderate. This finding is similar to that found by Barrett, Midgley, and Lovell [18]. They found that PL on the scapulae and the center of mass produced very large within-subject correlations with HR, but only negligible to moderate across-subject correlations between with HR [18]. Furthermore, we found that there is a similar difference between the AIMs on the wrist like the trunk, PL at the wrist and trunk had a stronger correlation with %HRR than MAD. The findings indicate that PL may be able to more sensitive to capture the change in external intensity which relates to internal intensity. Overall, accelerometers placement on wrist-based PL and shank-based regardless of PL and MAD are more ideal to monitor running exercise intensity. Trunk placements and wrist-based MAD are not recommended.

Previous studies have confirmed that PL is suitable for monitoring load in ball sports [16,17,31]. The finding of our study also supports this monitoring method. Further, we found that whether sensors place on the wrist, trunk, or shank, PL at those placements had moderate across-subjects correlation with %HRR (*r* = 0.463–0.628). Basketball is a sport involving whole-body, various movement types, and changing directions. In this study, the basketball exercise program included running (forward and backward), shuffle (left and right), jumping, shooting was used to evaluate the correlation between the %HRR and AIMs. This highly variable nature of basketball makes all accelerometer-placements have a similar correlation with %HRR; however, this nature also increases the negative impact on across-subjects (within-sport) relationship between the acceleration index and the intrinsic strength. Similar results also were found in MAD, MAD at all placements had a moderate correlation with %HRR (*r* = 0.403–604) under basketball. There is no obvious difference in the strength of correlation between the two AIMs with %HRR. If considering overall correlation with %HRR, accelerometers placement on shank appear to be preferred over wrist and trunk in basketball to correspond to the internal intensity demands.

For badminton, the result showed that both AIMs methods at racket-side shank had strong correlation (*r* = 0.782–793) with %HRR, followed by at the non-racket wrist (*r* = 0.587–0.621) and trunk-based PL (*r* = 0.608), and finally the trunk-based MAD (*r* = 0.314). Previous research found that the PL on the non-racket wrist (*r* = 0.796), lower back (*r* = 0.843), and racket-side ankle (*r* = 0.728) were strongly relate to TRIMP by HR [8]. However, the correlations of PL and MAD at the non-racket wrist and trunk and in this study were weaker. This discrepancy may be related to the placement position (upper back) and the different experimental movements. Six-point footwork with different movement tempos was adopted in the present study to assess the correlation between HR and ALLs, while the previous study performed different badminton technical skills, including backhand serve, net shot, six-point footwork, and jump smash. However, results seem to indicate that PL and MAD on the leg (shank or ankle) provide a more stable relationship with HR based-metrics (%HRR and TRIMP) for estimating exercise intensity. Based on our results, both PL and MAD can be used to monitor badminton exercise intensity and the racket-side shank placement is more ideal for racket sports, followed by wrist and trunk-based PL. Future research can focus on other racket sports, such as tennis and table tennis, to verify whether the racket-side shank is still a suitable sensor-placement location for evaluating the exercise intensity.

An evaluation of the correlation of the two AIMs on different placements and sports (Figure 6 and Figure 7) reveals that accelerometers placement on shank across all sport conditions produces the strongest within-sport and across-sport relationship with %HRR. This result could be because lower limbs activity is the main factor affecting the movement intensity in various sports. Lower limbs function to support the body and produce move and jump during various sports. In this study, lower limbs involved all movements (e.g., running, jumping, footwork) regardless of sports types (i.e., running, basketball, and badminton). Therefore, the AIMs at shank could well reflect the exercise intensity. Additionally, the evaluation indicates that a non-dominant wrist may be considered as a secondly ideal sensor-placement to capture both internal and external intensity under separate sports. However, MAD at the wrist is not universally applicable to all sports, the strength of correlation of wrist-based MAD and %HRR was weaker under running than PL, as well as the MAD at wrist did not increase with running speed from 12 to 15 kph. Therefore, if considering practical applications, PL on the non-dominant wrist seems a more generic method to monitor the intensity under different sports. People generally wear watches on non-dominant wrists, and most smart watches have built-in accelerometers for motion recognition and electrocardiogram or photoplethysmography sensors for HR monitoring. Therefore, it seems logical that a wrist-based device to simultaneously calculate external and internal loading may be ideal for sports applications. However, it should be noted that the PL or MAD calculations between different sports are not comparable because of the weak to moderate across-sports correlation with %HRR (*ρ* = 0.207–547). As illustrated by the data in Figure 4 and Figure 5, it is likely that the activity requirement of each sensor-placement location is different between different sports under the same intensity level. For example, under the intensity level 2, the PL at shank during running and badminton were respectively 2649 and 1732 AU (Figure 4), but the %HRR during running and badminton were 76 and 74 %HRR (Figure 3), respectively. Therefore, how to make AIMs comparable between different sports can be used as the direction of future research.

The limitation of this study was that all participants performed the same and selected sport-specific task at different times allotted for completing each task to distinguish different intensities under basketball and badminton. Although the selected exercise tasks were composed of sport-specific demands of basketball and badminton, respectively, it was still a controlled exercise task and was different from the practical exercise situation which has intermittent and rapid intensity changes. Further research is needed to determine the validity of PL and MAD at different sensor-placement in the practical exercise under different sports.

## 5. Conclusions

This study evaluated the relationship between %HRR and two AIMs (PL and MAD) with different sensor-placements locations and sport types. The strength of the correlations varied substantially by the different AIM methods, sensor-placements, and sport types. Overall, shank placement sites produced the strongest within-sport (across-subjects) correlation with %HRR in running, basketball, and badminton. Moreover, the PL-based calculation from an accelerometer on a non-dominant wrist seems may be a more generic method to use AIM for intensity and load monitoring in practical applications. However, it must be noted that the AIMs between different sports are not comparable due to weak to moderate across-sports correlation.

## Data Availability

The data presented in this study are available in the Supplementary Materials.

## Supplementary Materials

The following supporting information can be downloaded at: https://www.mdpi.com/article/10.3390/s22072583/s1.
