# Supplementary material for: Ideal Combinations of Acceleration-Based Intensity Metrics and Sensor Positions to Monitor Exercise Intensity under Different Types of Sports
Source: Sensors (Basel). 2022 Mar 28;22(7):2583. doi: 10.3390/s22072583 (PMC9003469; doi:10.3390/s22072583)
Supplement: Supplementary file 1 [file sensors-22-02583-s001.zip › sensors-1620265-supplementary.pdf]

Table S1. Change of %HRR, PL/min, and MAD among three exercise intensity levels under different sports

		%HRR	PL/min (AU / min)			MAD (mg)		
			Wrist	Trunk	Shank	Wrist	Trunk	Shank
Running	L1	58.87 ± 15.05†‡	1915 ± 263^{bc}	1198 ± 141†‡	2025 ± 150^{bc}	1106 ± 155^{bc}	717 ± 104^{b}	658 ± 79^{bc}
	L2	76.34 ± 13.46*‡	2335 ± 319^{ac}	1504 ± 176*‡	2649 ± 211^{ac}	1199 ± 178^{a}	761 ± 84^{a}	798 ± 65^{ac}
	L3	89.34 ± 11.86*†	2700 ± 289^{ab}	1817 ± 162*†	3372 ± 292^{ab}	1247 ± 164^{a}	738 ± 77	943 ± 68^{ab}
Basketball	L1	67.76 ± 18.07^{bc}	1306 ± 219^{bc}	915 ± 117†‡	1573 ± 166†‡	848 ± 101^{bc}	510 ± 52^{bc}	519 ± 40^{bc}
	L2	74.52 ± 17.74^{ac}	1524 ± 243^{ac}	1076 ± 102*‡	1822 ± 133*‡	976 ± 112^{ac}	591 ± 59^{ac}	592 ± 25^{ac}
	L3	80.05 ± 15.22^{ab}	1690 ± 209^{ab}	1215 ± 96*†	2095 ± 187*†	1052 ± 91^{ab}	629 ± 64^{ab}	653 ± 44^{ab}
Badminton	L1	65.37 ± 14.43^{bc}	1064 ± 270^{c}	966 ± 160^{c}	1625 ± 230^{bc}	681 ± 122†‡	536 ± 80^{bc}	524 ± 55^{bc}
	L2	73.60 ± 12.86^{ac}	1150 ± 313^{c}	1030 ± 183^{c}	1732 ± 284^{ac}	727 ± 144*‡	565 ± 87^{ac}	553 ± 64^{ac}
	L3	82.21 ± 10.66^{ab}	1307 ± 337^{ab}	1157 ± 184^{ab}	1941 ± 322^{ab}	788 ± 151*†	593 ± 87^{ab}	595 ± 58^{ab}

L=intensity level.

Bonferroni post hoc test: significantly different than ^{a}L1, ^{b}L2, and ^{c}L3, $p<.05$.

Wilcoxon signed rank test: significantly different than *L1, †L2, and ‡L3, $p<.05$.

Table S2. The within-sport Pearson (r) and across-sports Spearman (ρ) correlation coefficient for %HRR and PL as well as %HRR and MAD.

		Running		Basketball		Badminton		Across-sports	
		r	p	r	p	r	p	ρ	p
Wrist	PL	0.762	0.000**	0.536	0.000**	0.587	0.000**	0.342	0.000**
	MAD	0.448	0.003**	0.603	0.000**	0.621	0.000**	0.322	0.000**
Trunk	PL	0.451	0.003**	0.463	0.002**	0.608	0.000**	0.390	0.000**
	MAD	0.065	0.684	0.403	0.008**	0.314	0.043*	0.207	0.020*
Shank	PL	0.777	0.000**	0.628	0.000**	0.793	0.000**	0.547	0.000**
	MAD	0.778	0.000**	0.604	0.000**	0.782	0.000**	0.484	0.000**

*$p< .05$; **$p<.01$
